# Case Report: A case of giant emphysematous bullae successfully treated with percutaneous aspiration and sclerotherapy for staged reduction of pulmonary bullae

**DOI:** 10.3389/fmed.2025.1693942

**Published:** 2025-10-08

**Authors:** Chao Li, Xiao Hu, Gang Jiang, Yong Liang Jiang

**Affiliations:** ^1^Department of Respiratory Medicine, Hunan Provincial People's Hospital (The First-Affiliated Hospital of Hunan Normal University), Changsha, Hunan, China; ^2^Clinical Medicine Research Center For Respiratory Rehabilitation in Hunan Province, Changsha, China

**Keywords:** emphysema, pulmonary bullae, percutaneous sclerotherapy, polidocanol, minimally invasive procedure

## Abstract

Giant emphysematous bullae (GEB) in COPD patients typically require high-risk surgery, with limited and minimally invasive alternatives. This report describes a 70-year-old man with COPD GOLD 3 and bilateral GEB (dominant bulla 8.5 cm× 6.2 cm) who underwent a novel percutaneous serial sclerotherapy protocol: Under CT guidance, a puncture needle was inserted into the bulla cavity on 3 consecutive days, with daily instillation of polidocanol (total 30 mL) and attempted air aspiration (successfully retrieving 1,000 mL of gas on day 3). No pneumothorax, desaturation, or bleeding occurred peri-procedurally. A follow-up CT scan at 3 months demonstrated>70% bullae volume reduction with lung re-expansion, correlating with significant clinical improvement. This first-reported percutaneous aspiration and sclerotherapy for staged reduction of pulmonary bullae (PASS) approach offers a safe, effective, minimally invasive option for GEB management in surgically high-risk COPD patients, warranting further validation of the protocol.

## Introduction

Giant emphysematous bullae (GEB), characterized by one or more air-filled spaces occupying at least one-third of a hemithorax and displacing normal lung parenchyma, represent a severe complication of chronic obstructive pulmonary disease (COPD) with an estimated incidence of 2 per million individuals (1). These pathological structures progressively compress functional lung tissue, leading to debilitating dyspnea, hypoxemia, and increased mortality risk (2). Traditional management relies on surgical bullectomy, which—despite being definitive—carries substantial morbidity (15–30%), including prolonged air leaks, respiratory failure, and mortality in high-risk COPD populations (3). While bronchoscopic lung volume reduction techniques (e.g., endobronchial valves and coils) have emerged as minimally invasive alternatives (4), their efficacy is limited by anatomical prerequisites: Valves require intact interlobar fissures (>85% completeness) and target lobe absence of collateral ventilation (5), whereas coil deployment necessitates heterogeneous emphysema distribution (6). Percutaneous approaches offer theoretical advantages for peripheral bullae that are inaccessible to bronchoscopy; however, prior attempts with single-session sclerotherapy (ethanolamine oleate, autologous blood) have yielded inconsistent results due to inadequate cavity collapse or inflammation induction (7–9). Polidocanol—a detergent sclerosing agent widely used in venous malformation therapy—induces fibroblast proliferation and pleural fibrosis through protein denaturation and cytokine-mediated inflammation (10), but its application in GEB remains unexplored. The critical knowledge gap lies in developing a standardized percutaneous protocol ensuring sustained bullae reduction without procedure-related complications (pneumothorax, infection). Herein, we present the first documented case utilizing serial CT-guided polidocanol instillation over three consecutive days, resulting in a significant volume reduction in a surgically ineligible COPD patient.

## Case

**Figure 1:**
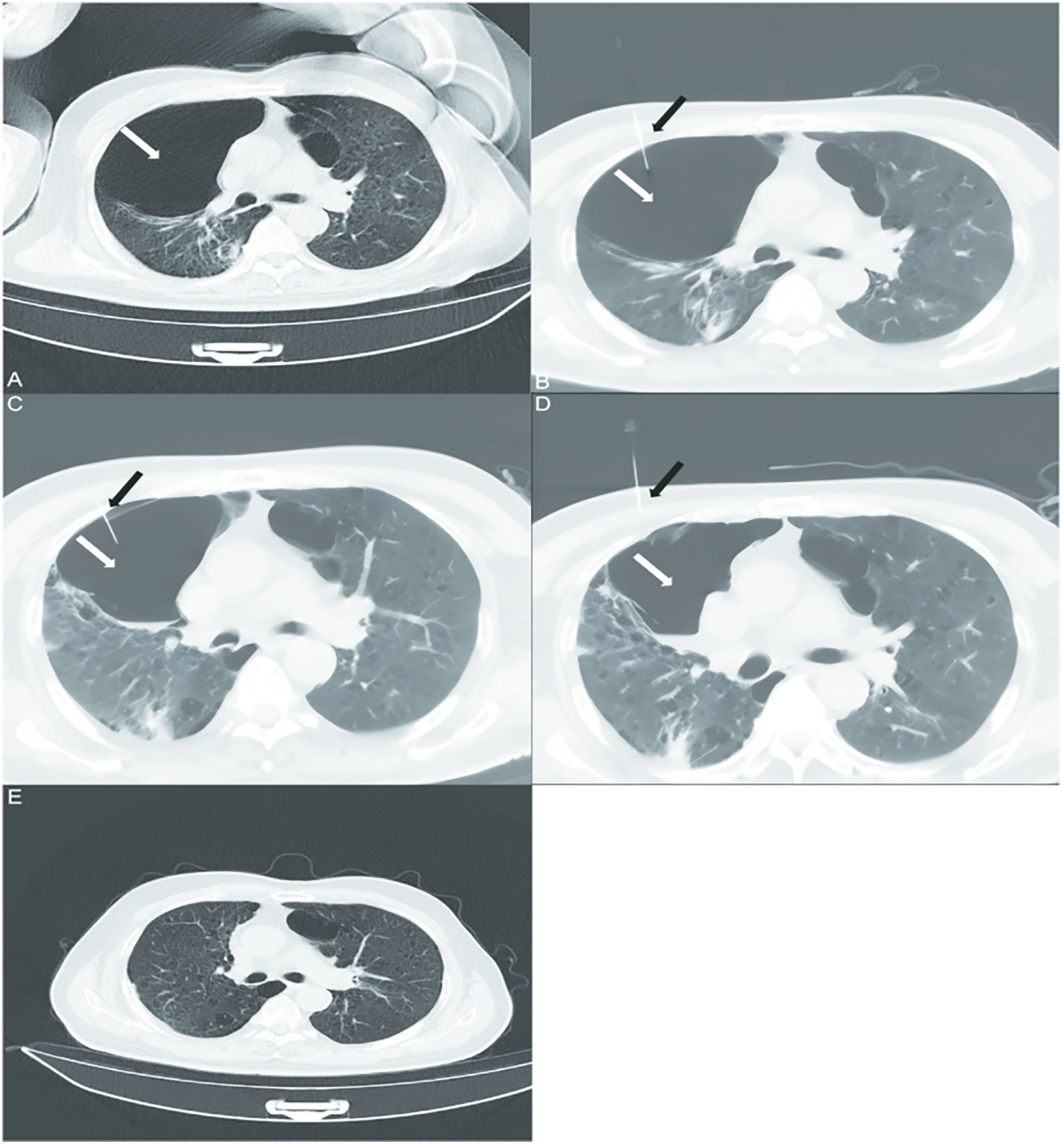
Axial computed tomography scans of the chest in patients with bilateral emphysema and alveoli before and after treatment with percutaneous bullae reduction surgery. **(A)** Chest CT shows emphysema and pulmonary bullae in both lungs(white arrows). **(B–D)** A puncture needle (black arrows) was inserted into the patient’s lung cavity on 3 consecutive days, and the air was aspirated and injected with polidocanol. **(E)** On follow-up chest CT at 3 months of treatment, the patient’s pulmonary alveoli showed significant improvement.

A 70-year-old man with severe COPD (GOLD stage 3, FEV1 < 48% predicted) and a past medical history significant for hypertension and coronary artery disease status post percutaneous coronary intervention 5 years prior, and 40-pack-year smoking history presented to hospital with recurrent cough, productive sputum, and progressive dyspnea worsening over 12 months despite maximal medical therapy (long-acting muscarinic antagonist/long-acting agonist, inhaled corticosteroids, home oxygen at 2 L/min). Physical examination findings were consistent with severe COPD and the presence of large bullae. There was no significant family history of genetic lung disease (e.g., alpha-1 antitrypsin deficiency) or other relevant psychosocial factors beyond the reported smoking history. Baseline high-resolution computed tomography (HRCT) revealed bilateral giant emphysematous bullae with the dominant lesion measuring 8.5 cm× 6.2 cm in the right upper lobe, compressing adjacent parenchyma (Figure 1A). Following multidisciplinary assessment, the patient—deemed high-risk for surgery due to severe air trapping and chronic hypercapnia—underwent a novel percutaneous aspiration and sclerotherapy for staged reduction of pulmonary bullae (PASS) protocol (Table 1) on the first day, under local anesthesia and CT guidance (Philips, Precedence 16, Netherlands), a puncture needle was directly inserted into the patient’s lung cavity, and polidocanol was injected into the cavity to ensure adequate contact with the pulmonary bullae (Figure 1B). The patient was rotated through prone, supine, and lateral decubitus positions to maximize sclerosing agent contact with bulla walls. On the second day, when the puncture needle was inserted again, an attempt was made to aspirate gas, but it proved challenging, and no reduction in the size of the bullae was observed. The injection of polidocanol into the cavity continued (Figure 1C). On the third day, gas aspiration was successful after inserting the puncture needle into the lung cavity, and polidocanol injection continued (Figure 1D). Peri-procedural monitoring showed stable oxygen saturation (SpO_2_ 92–95% on 2 L/min nasal cannula) without pneumothorax or hemodynamic compromise. Post-procedure chest radiographs confirmed the absence of iatrogenic complications. At 3-month follow-up, HRCT demonstrated >70% volume reduction of the dominant bulla (residual size 2.8 cm× 3.1 cm) with re-expansion of compressed lung tissue (Figure 1E), correlating with significant symptomatic improvement: mMRC dyspnea scale decreased from 4 to 2, 6-min walking distance increased from 182 m to 310 m, and chronic oxygen therapy was discontinued. Subjectively, the patient reported a marked improvement in his ability to perform activities of daily living independently and expressed high satisfaction with the procedural outcome.

**Table 1:** PASS protocol: daily procedure summary.

Phase	Time point	Key procedures and observations
Treatment phase	Day 1	CT-guided puncture and injection- Instillation of 10 mL of polidocanol - Patient positional rotation - Attempted aspiration (unsuccessful)
	Day 2	CT-guided puncture and injection- Instillation of 10 mL of polidocanol- Attempted aspiration (unsuccessful)
	Day 3	CT-guided puncture and injection- Instillation of 10 mL of polidocanol- Successful aspiration (1,000 mL of gas extracted)
Monitoring and outcomes	Peri-procedure	Stable oxygen saturation (SpO₂ 92–95%)- No complications such as pneumothorax or bleeding
	3-month Follow-up	>70% reduction in bulla volume- mMRC dyspnea scale decreased from 4 to 2–6-min walking distance (6MWD) increased from 182 m to 310 m- Discontinuation of oxygen therapy

## Discussion

**Figure 2:**
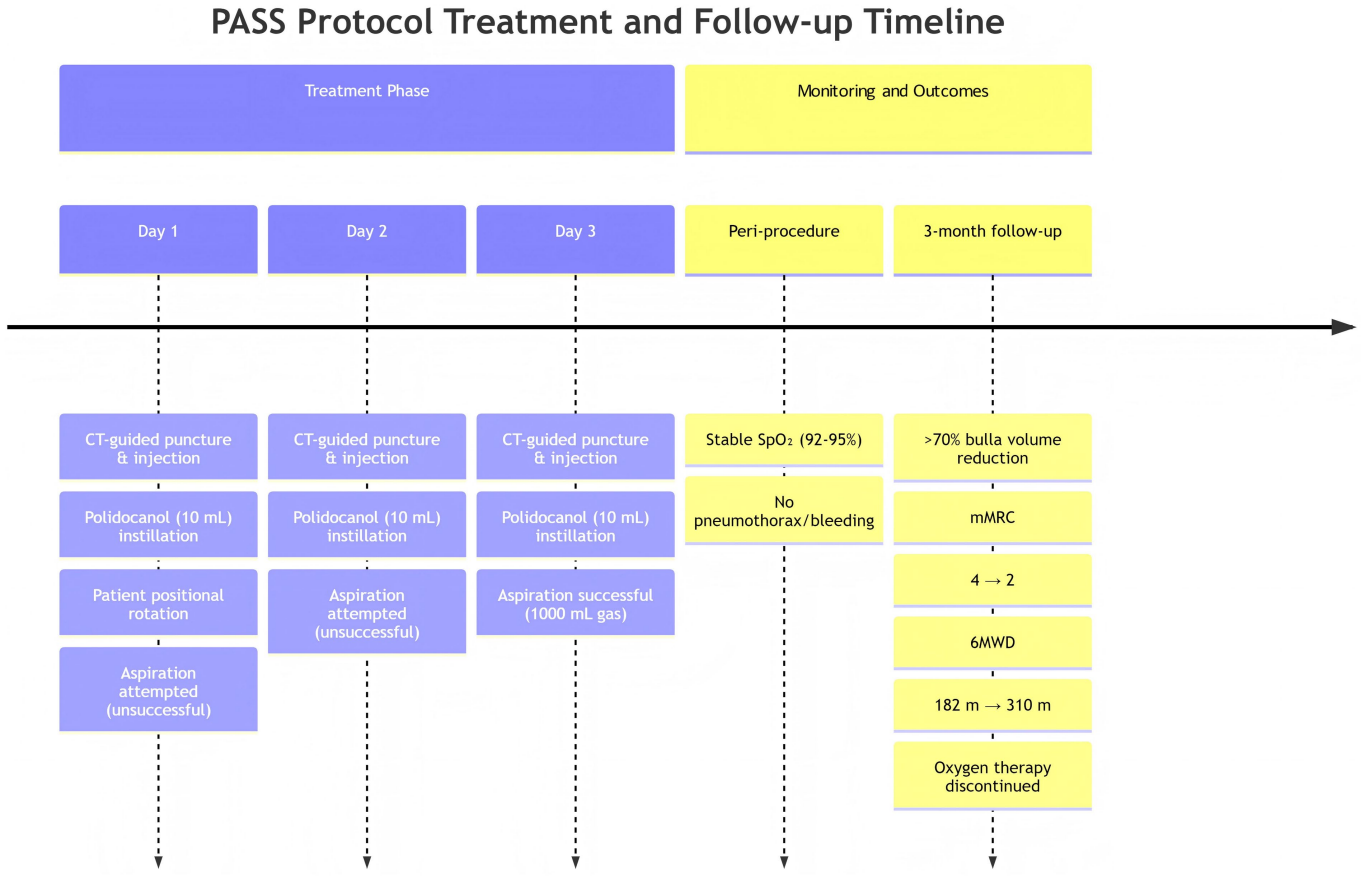
PASS protocol: timeline chart for PASS protocol.

Management of symptomatic GEB in advanced COPD patients who are suboptimal candidates for surgery presents a significant therapeutic dilemma. While bronchoscopic lung volume reduction techniques offer a minimally invasive alternative, their application is limited by stringent anatomical criteria, which exclude a substantial proportion of patients (3). Percutaneous sclerotherapy emerged as a potential solution for peripheral bullae, yet historical attempts with single-session agents often yielded suboptimal outcomes due to inadequate cavity collapse or reperfusion (6, 7). The PASS protocol was developed to directly address these limitations through a novel, multi-mechanistic approach (see Figure 2).

The decision to intervene in GEB remains nuanced. There is consensus that asymptomatic patients, even with large bullae, can often be managed conservatively with observation, as the risks of intervention may outweigh the benefits. Surgical bullectomy or minimally invasive techniques are typically reserved for symptomatic patients who exhibit disabling dyspnea, functional impairment, or complications directly attributable to the bullae, such as recurrent pneumothorax, infection within the bulla, or significant compression of adjacent viable lung parenchyma leading to hypoxemia. Ideal surgical candidates are those with large, dominant peripheral bullae that compress healthy lung tissue, preserved lung function in the non-bullous regions, and an acceptable cardiopulmonary reserve to tolerate the procedure. Our patient, who suffered from progressive dyspnea (mMRC4), functional limitations, and radiographic evidence of lung compression, clearly fell into this symptomatic category yet was deemed unfit for surgery due to his high-risk profile, thus making him an ideal candidate for the PASS protocol. The PASS protocol described here addresses these limitations through several key innovations. First, the use of serial instillation of polidocanol over 3 consecutive days exploits the compound’s fibrogenic properties. Polidocanol, a detergent sclerosant, induces protein denaturation and robust cytokine-mediated inflammation (notably sustained TGF-β1 elevation), leading to fibroblast proliferation and accelerated collagen deposition within the bulla wall (8, 9). Histological studies confirm collagen matrix formation can commence within 72 h of polidocanol exposure (10)—significantly faster than talc (7–10 days) (11). Second, the incorporation of staged aspiration is critical. Attempted aspiration on day 2 facilitated initial decompression, while successful aspiration of 1,000 mL of gas on day 3 capitalized on the evolving inflammatory process and weakened the bulla wall, preventing pressure re-equilibration and promoting sustained collapse. Third, deliberate positional rotation of the patient after each instillation ensured circumferential contact of polidocanol with the bulla epithelium, maximizing the sclerosing effect throughout the cavity—a factor often overlooked in prior techniques.

The selection of a 72-h interval between procedures represents a strategic balance. It allows sufficient time for peak cytokine release (occurring approximately 48 h post-sclerotherapy) (8) to initiate robust inflammation and fibroblast recruitment, yet is brief enough to prevent significant re-epithelialization of the damaged bulla lining. This contrasts with staged talc pleurodesis protocols, which use longer 7-day intervals (11), reflecting the differing pharmacodynamics of the agents. Importantly, despite three transthoracic punctures, the procedure demonstrated an excellent safety profile. The absence of pneumothorax likely relates to the inherent characteristics of giant bullae: Their thick walls provide structural integrity, while the use of a small-gauge needle minimizes parenchymal trauma. This compares favorably to the 28% pneumothorax rate reported for standard CT-guided lung biopsies (12). Furthermore, no desaturation, bleeding, or infection occurred, underscoring the suitability of PASS for surgically high-risk patients, such as those with chronic hypercapnia or severe air trapping.

The clinical and radiological outcomes in this case were compelling. The >70% volume reduction of the dominant bulla at 3 months, confirmed by HRCT, translated into significant symptomatic improvement: a reduction in mMRC dyspnea score from 4 to 2, a near doubling of 6-min walking distance (182 m to 310 m), and liberation from chronic oxygen therapy. This degree of efficacy surpasses that reported in earlier percutaneous sclerotherapy trials. For instance, Saji et al. observed only a 38% volume reduction using fibrinogen-thrombin (6), while Snell et al. noted frequent reperfusion of cavities treated with single-session polidocanol (7). The staged, multi-mechanistic approach to PASS appears crucial for achieving durable collapse.

This report describes the first successful application of the PASS protocol in a single patient. While promising, the efficacy and safety require validation in larger, prospective cohorts with long-term follow-up to assess durability beyond 3 months and identify potential late complications (e.g., infection within the sclerosed cavity). Optimizing the protocol parameters (e.g., ideal sclerosing agent concentration/volume, optimal number of stages, precise aspiration timing) warrants further investigation. Patient selection criteria also need refinement; ideal candidates likely have large, tense, peripheral bullae with thick walls on CT, a compressive functional lung, and no active infection.

## Conclusion

The PASS protocol—combining CT-guided percutaneous serial polidocanol sclerotherapy, staged aspiration, and positional rotation—represents a promising, truly minimally invasive therapeutic breakthrough for managing giant emphysematous bullae in high-risk COPD patients unsuitable for surgery or bronchoscopic techniques. This case demonstrates its potential for achieving significant and rapid bulla volume reduction with consequent clinical improvement and an excellent procedural safety profile. Larger clinical trials are warranted to confirm these findings and establish PASS as a viable standard option for this challenging patient population.

## Data Availability

The original contributions presented in the study are included in the article/supplementary material, further inquiries can be directed to the corresponding author.
